# Will Septal Correction Surgery for Deviated Nasal Septum Improve the Sense of Smell? A Prospective Study

**DOI:** 10.1155/2015/496542

**Published:** 2015-09-29

**Authors:** Neelima Gupta, P. P. Singh, Rahul Kumar Bagla

**Affiliations:** Department of Otorhinolaryngology, University College of Medical Sciences and GTB Hospital, Delhi 110095, India

## Abstract

*Background and Objectives*. Nasal obstruction due to deviated nasal septum is a common problem bringing a patient to an otorhinolaryngologist. Occasionally, these patients may also complain of olfactory impairment. We proposed to study the effect of septal deviation on the lateralised olfactory function and the change in olfaction after surgery of the septum (septoplasty).* Methods*. Forty-one patients with deviated nasal septum were evaluated for nasal airflow, olfactory score, and nasal symptomatology. Septoplasty was done under local anesthesia. Pre- and postoperative olfactory scores, airflow and olfactory scores, and nasal symptomatology and olfactory scores were compared and correlated.* Results*. The range of preoperative composite olfactory score (COS) on the side of septal deviation was 4–14 (mean 7.90 ± 2.234) and on the nonobstructed side was 9–18 (mean 14.49 ± 2.378). Severity of deviated nasal septum and preoperative COS of diseased side were correlated and the correlation was found to be significant (rho = −0.690, *p* = 0.000 (<0.001)). The preoperative mean COS (7.90 ± 2.234) was compared with the postoperative mean COS (12.39 ± 3.687) and the improvement was found to be statistically significant (*p* = 0.000 (<0.001)).* Conclusion*. We found improvement in olfactory function in 70.6% patients after surgery, no change in 20.1%, and reduced function in 7.6%. With the limitation of a small sample size and a potential repeat testing bias, we would conclude that correction of nasal septal deviation may lead to improvement in sense of smell.

## 1. Introduction

Nasal obstruction is one of the most common problems bringing a patient to an otorhinolaryngologist's office, and septal deviation is a frequent structural etiology. Though nasal obstruction is the primary complaint in these patients, occasionally they may also complain of olfactory impairment. Higher olfactory thresholds as well as compromised olfactory identification have been documented on the deviated side of a septal deviation. Studies have shown that the structure of nasal cavity determines the pattern of airflow through the nose, thus affecting the number of odorant molecules transported to the olfactory epithelium. Leopold studied the relationship between nasal anatomy and human olfaction and found a relationship between changes in the structure of the upper nasal cavity and changes in olfactory ability [1]. Several other studies have focused on the relationship between the intranasal airflow and olfactory function [2, 3].

Damm et al. [2] assessed the intranasal volume and its relation to olfactory function in normosomic subjects using MRI scans. They found significant correlations between odor threshold measurements and volumes of the segment in the upper meatus directly below the cribriform plate and the anterior segment of inferior meatus.

Septoplasty most often improves the nasal respiratory airflow. Though studies have assessed the outcome of septal surgery in relation to nasal airflow, not many studies have focused on the change in olfaction following septal surgery. Also barring isolated studies [4], most studies have not directly correlated the uninasal airflow measurements and olfactory function before and after septal surgery. We proposed to study the effect of septal deviation on the lateralised olfactory function and correlate it with the visibility of the olfactory cleft and extent of posterior septal deviation. We also studied the change in olfaction after surgery of the septum. There are no reports from India about the effect of septal surgery on the olfactory function and in the presence of western reports about decrease in olfactory function following surgery due to direct trauma or vascular compromise to the olfactory epithelium [5]; it becomes imperative to investigate all septoplasty patients for this important aspect of nasal function.

## 2. Material and Methods

The study received approval by the institutional ethical committee. Forty-one patients presenting with nasal obstruction due to deviated nasal septum and impaired sense of smell were included in the study over one year. The age group was 15 to 45 years. Patients suffering from acute rhinitis, chronic rhinosinusitis, atrophic rhinitis, granulomatous diseases of nose, and nasal masses and patients having past history of nasal surgery were excluded from the study.

All patients were evaluated in detail with a history which included short form nasal questionnaire [6] for nasal symptomatology and visual analogue score (from 0 to 5) for subjective grading of their sense of smell, with grade of “5” being very poor and grade “0” being almost normal. Using short form nasal questionnaire (SFNQ), the symptoms graded were nasal obstruction (degree and duration), nasal stuffiness, excess mucus production, postnasal drip, snoring, and overall nasal symptoms on a scale of 0–4. For example, score 0 was given when there was no nasal obstruction and score 4 was given when the nasal obstruction was reported as very severe. The patients underwent test for olfaction, routine ENT examination with focus on anterior rhinoscopy, anterior rhinomanometry using nasal olives, and nasal endoscopy using a 0-degree nasal endoscope. The septal deviation was graded on anterior rhinoscopy as grade 1, mild septal deviation; grade 2, septum close to inferior turbinate; and grade 3, septum touching the inferior turbinate/lateral nasal wall.

Each side was graded separately and scores from each side were taken as “right nostril” and “left nostril” individual scores. The septal deviation and the visibility of olfactory cleft on endoscopy were recorded. According to the side of septal deviation, the nasal cavities were divided into diseased (obstructed) and nondiseased side.

Patients were worked up for septoplasty under local anaesthesia. Septal surgery was performed in which the deviated nasal septum was straightened with preservation of cartilaginous and bony parts of septum as much as possible. Nasal pack was removed after 48 hours and patients were kept under weekly follow-up.

Olfactory test, rhinomanometry, SFNQ, and VAS were repeated after four weeks of surgery.

### 2.1. Olfactory Test Methodology [7, 8]

The test comprised of two components: the olfactory threshold and identification.

#### 2.1.1. Threshold Testing

The threshold test employed 1-butanol as the test odorant. The test kit contained nine glass bottles each containing ~20 mL of test solution (solutions one to nine) and another identical glass bottle filled with ~20 mL of sterile water. The 1-butanol solution was diluted by successive factors of three, the highest concentration being 4%, designated as solution one while the lowest concentration is 0.00061%, designated as solution nine. Participants received two bottles at a time, one with sterile water and one with odorant. The test begins with the weakest solution in an ascending order of concentration to avoid desensitization. The lowest concentration of odorant that the patient correctly identifies on four successive occasions was defined as the threshold. Scores of one to nine were given depending on the lowest concentration of solution successfully identified. If the solution with the highest concentration was not identified, a score of zero was given. After determination of the threshold in one nostril, testing was done for the other nostril in the similar manner.

#### 2.1.2. Odor Identification Testing

The odorant substances were kept in opaque plastic bottles and the patient's eyes were covered when the bottles were presented to them. To perform the test, the cap was removed by the examiner for approximately 3 seconds, and the tip of the bottle was placed approximately 2 cm in front of the nostril and patient was asked to sniff normally without any force. There was an interval of at least 30 seconds between successive presentations to prevent olfactory desensitization [9]. Patients were asked to choose from a list of four choices for each substance presented. Ten items were presented in random order for monorhinic smelling [10]. To restrict the stimulus to one nostril, the participant was asked to hold the other nostril closed. The total odor identification score was calculated by adding the number of substances correctly identified. The total score of the threshold test and the odor identification test was taken up as the combined olfactory score (COS) for the nostril being tested.

Substances for odor identification were asafoetida (heeng), naphthalene balls (moth balls), garlic (lahsun), Vicks VapoRub, rose water, cinnamon (dal chini), sandalwood oil, cardamom (elaichi), clove oil (laung), lemon, coffee, mint (pudina), camphor (kapur), and cumin seeds (jeera).

The combined olfactory score estimation using the threshold testing and odor identification testing has been previously validated in the Indian population [7]. The odors used for identification testing purposes have been selected after surveys and pilots and are according to Indian cultural preferences. The odors used in CCCRC are Johnson baby powder, chocolate, cinnamon, coffee, mothballs, peanut butter, Ivory bar soap, ammonia, Vicks VapoSteam, and wintergreen [10]. Since a few of these odors are not familiar to the Indian population, so we have used our previously validated “I-Smell” test.

For an objective assessment of nasal airflow we used anterior rhinomanometry. It is generally agreed that rhinomanometry with synchronous recording of flow rate and pressure drop across the nasal cavity during spontaneous breathing is the preferable and most reliable method for measuring nasal patency [11].

The HOMOTH-400 Rhinomanometer was used and Active Anterior Rhinomanometry (AAR) was done using nasal olives. The value of nasal airflow (NAF) at 150 pascals was taken for all assessments of nasal airflow.

### 2.2. Data Collection and Analysis

All relevant data were tabulated and systematically analysed using SPSS 17 statistical software. Wilcoxon's Sign Rank Test was used to compare the preoperative and postoperative olfactory score, short form nasal questionnaire score, and VAS score of olfaction. Spearman's/Pearson's correlation was used for correlation between olfactory score and rhinomanometry values both preoperative and postoperative and correlation between short form nasal questionnaire (SFNQ) and olfactory score. Paired *t*-test was used for comparing preoperative and postoperative flow rate values on rhinomanometry. *p* value <0.05 was considered significant.

## 3. Results

Over a period of 1 year, a total of 41 subjects with deviated nasal septum on presentation were prospectively recruited. The age of the subjects ranged from 15 to 45 years (mean 25.51 years). There were 27 males and 14 females with a male-to-female ratio of 1.9 : 1.

The mean preoperative SFNQ score was 16.20 ± 3.494. The mean preoperative VAS score was 3.27 ± 0.633.

The olfactory cleft on diseased side was visible in 12 patients and on nondiseased side it was visible in 31 patients. On diseased side, posterior septal deviation was present in 10 patients, while it was absent in all patients on nondiseased side.

The range of COS on the diseased side was 4–14 (mean 7.90 ± 2.234); and on the nondiseased side it was 9–18 (mean 14.49 ± 2.378). Since we classified the side with the deviation of septum as the “diseased side,” we further analyzed the parameters recorded on the obstructed/diseased side.

The severity of deviated nasal septum (DNS) on anterior rhinoscopy and preoperative COS of diseased side were correlated using Spearman's correlation and the correlation was found to be significant (rho = −0.690, *p* = 0.000 (<0.001)). The more severe the deviation was the less the COS was. The distribution of preoperative COS was compared with the olfactory cleft being visible or not visible using Mann-Whitney *U* test. The comparison was not significant (*p* = 0.134). Similarly the comparison between COS and posterior septal deviation being present or absent was insignificant (*p* = 0.042).

The preoperative mean score of SFNQ (16.20 ± 3.494) changed to postoperative mean score of SFNQ (7.78 ± 3.848), implying that there was decrease in the symptoms assessed on SFNQ after 4 weeks of follow-up and the improvement was found to be statistically significant (*p* = 0.000 (<0.001)).

The preoperative mean VAS (3.27 ± 0.633) was compared with postoperative mean VAS (1.56 ± 0.709) and the improvement was found to be statistically significant (*p* = 0.000 (<0.001)).

**Figure 1:**
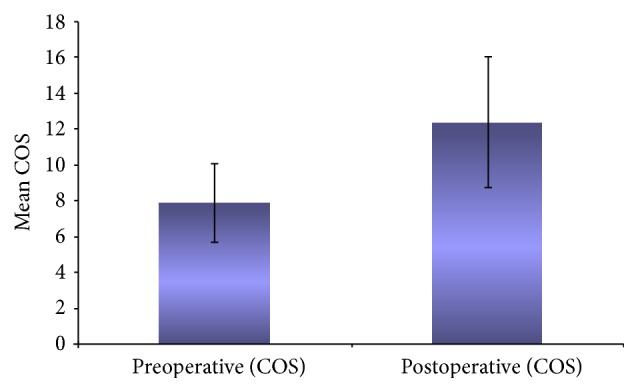
Preoperative and postoperative mean composite olfactory score (COS).

The preoperative mean COS (7.90 ± 2.234) on the diseased side was compared with the postoperative mean COS (12.39 ± 3.687), using the Wilcoxon Signed Rank Test and the improvement was found to be statistically significant (*p* = 0.000 (<0.001)). The improvement is shown in Figure 1.

All values of various parameters evaluated before and after surgery are shown in Table 1. The improvement in mean NAF after surgery was found to be statistically significant, both during inspiration and expiration (*p* < 0.001).

The preoperative VAS (3.27 ± 0.633) and preoperative COS (7.90 ± 2.234) of diseased side were correlated using Spearman's correlation and the correlation was found to be statistically significant (rho = −0.493, *p* = 0.001 (<0.001)), implying therefore that the objective olfaction test values correlated with the subjective sense of smell and there is a lateralised difference in olfactory score on the deviated side. The postoperative VAS and postoperative COS values of the diseased side showed a statistically significant inverse correlation (rho = −0.713, *p* = 0.000 (<0.001)). This indicates that after septal surgery, the subjective sense of smell improved (decreased mean VAS) and the composite olfactory score improved (increased mean COS) on the septal deviation side.

The preoperative COS (7.90 ± 2.234) correlated with the preoperative SFNQ (16.20 ± 3.494) scores significantly (rho = −0.436, *p* = 0.004 (<0.005)). More severe nasal symptoms were therefore found to be associated with decreased olfaction. And decrease in symptoms was associated with improved COS postoperatively (rho = −0.497, *p* = 0.001 (<0.001)).

The pre- and postoperative mean nasal air flow scores during inspiration and expiration were correlated with pre- and postoperative COS and the correlations were found to be significant. All correlations are shown in Table 2.

## 4. Discussion

The changes in olfactory ability following the correction of septal deviation is one way of evaluating how structural changes in nasal anatomy relate to olfactory ability. Septal surgery produces a change in nasal airflow and also leads to improvement in the patient's olfactory abilities in majority of cases.

In our study, preoperatively, there was low composite olfactory score (COS) on the deviated nasal septum side. Objective assessments of preoperative nasal airflow correlated with the composite olfactory scores, showing that decreased airflow was associated with low COS. Previous study by Fyrmpas et al. also concluded that significant nasal septal deviation impairs the ability to identify a smell from the obstructed nostril [12].

In our set of patients, following septal surgery improvement of olfactory function was seen in 29 (70.6%); no change was seen in 5 (20.1%); and reduced olfactory function was observed in 3 (7.3%) patients. Four patients were lost to follow-up. In contrast Pade and Hummel [13] reported improvement in olfaction in 13%, no change in 81%, and decreased function in 7% of patients after septal surgery. They observed that patients exhibiting a postoperative decrease of olfactory function had significantly higher preoperative olfactory scores than patients who experienced improvement.

Different studies have reported variable outcomes of septal surgery on olfaction.

C. N. Stevens and M. H. Stevens [14] measured the olfactory thresholds before and after surgery in 100 patients. The primary surgical procedure of 63 patients was septoplasty, of 24 septorhinoplasty, of 3 turbinate reduction, and of 10 polypectomy. The authors concluded that all the surgical procedures improved the olfactory function; however, the data for each type of operation was not provided separately.

Pfaar et al. [15] in his landmark study produced three major findings: (1) before surgery odor thresholds were related to nasal obstruction, (2) during the postoperative period a significant decrease of odor discrimination was found, whereas there was no change in odor thresholds and odor identification, and (3) no significant change of olfactory function was reported after surgery. This type of inference was also drawn by Doty et al. [16] and Kimmelman [5], who reported a small proportion of patients (1.1%) showing anosmia 2–4 weeks after surgery.

These observations differ from what we observed in our study as we observed more cases with postoperative improvement. Also similar to our study, Damm et al. [17] in their study found improvement in odor identification and odor discrimination in 80% and improvement in odor threshold in 54% patients after septoplasty in combination with partial inferior turbinectomy. Our observations are limited on the aspect of change in odor discrimination because we did not score odor discrimination.

Kimmelman [5] administered the UPSIT before and after septoplasty to 34 patients. The mean UPSIT scores of these largely normally functioning patients were essentially equivalent before and after the operation. However, in 15 rhinoplasty patients a small but statistically significant increase in performance was noted postoperatively. It was observed that since no control group was tested, a bias due to repeated testing may have accounted for the improvement after intervention. This is also a limitation of our study because we also did not have a control group.

Results of these various studies and our study suggest that septal surgery produces a variable outcome in terms of olfactory ability. Improvement in sense of smell can largely be attributed to an improvement in nasal airflow leading to a sense of improved ability to smell substances. A larger number of subjects with varying degrees of septal deviation, division of deviation into anatomical segments, and inclusion of a control group for lateralised difference in olfaction may bring out a better supported conclusion. Since we used composite olfactory score and not detection threshold and odor identification score separately we cannot comment on effect of septal deviation on suprathreshold olfactory function. However, we would like to conclude that even though the septal surgery is performed in an area remote from the olfactory epithelial area, changes in nasal airflow and intranasal volume can change the olfactory function of an individual.

## Figures and Tables

**Table 1:** Mean ± standard deviation of variables studied (on the obstructed side).

Variable	Preoperative	Postoperative
COS	7.90 ± 2.234	12.39 ± 3.687
VAS	3.27 ± 0.633	1.56 ± 0.709
SFNQ	16.20 ± 3.494	7.78 ± 3.848
NAF (inspiration) (cm^3^/sec)	219.24 ± 87.296	300.37 ± 143.652
NAF (expiration) (cm^3^/sec)	252.40 ± 84.986	352.05 ± 164.484

**Table 2:** Correlation matrix between combined olfactory score on the obstructed side of the nose and rest of the variables, both pre- and postoperatively.

Variable	Preop COS (7.90 ± 2.234)	Postop COS (12.39 ± 3.687)
VAS	Rho = −0.493 (*p* = 0.001)	Rho = −0.713 (*p* = 0.000)
SFNQ	Rho = −0.436 (*p* = 0.004)	Rho = −0.497 (*p* = 0.001)
NAF (inspiration)	Rho = 0.440 (*p* = 0.004)	Rho = 0.739 (*p* = 0.000)
NAF (expiration)	Rho = 0.512 (*p* = 0.001)	Rho = 0.771 (*p* = 0.000)
